# On an open question of V. Colao and G. Marino presented in the paper “Krasnoselskii–Mann method for non-self mappings”

**DOI:** 10.1186/s40064-016-2977-8

**Published:** 2016-08-11

**Authors:** Meifang Guo, Xia Li, Yongfu Su

**Affiliations:** 1 Department of Mathematics and Sciences, Hebei GEO University, Shijiazhuang, 050031 China; 2 Department of Mathematics, Tianjin Polytechnic University, Tianjin, 300387 China

**Keywords:** Hilbert space, Nonexpansive mapping, Non-self mapping, Mann iterative scheme, Inward condition, Weak convergence, Strong convergence, 47H05, 47H09, 47H10

## Abstract

Let *H* be a Hilbert space and let *C* be a closed convex nonempty subset of *H* and $T : C\rightarrow H$ a non-self nonexpansive mapping. A map $h : C\rightarrow R$ defined by $h(x) := \inf \{\lambda \ge 0 : \lambda x+(1-\lambda )Tx \in C \}$. Then, for a fixed $x_0 \in C$ and for $$\begin{aligned}{\alpha _0} = \max \left\{ {\frac{1}{2},h({x_0})} \right\}\end{aligned}$$, Krasnoselskii–Mann algorithm is defined by $x_{n+1}=\alpha _n+(1-\alpha _n)Tx_n,$ where $\alpha _{n+1}=\max \{\alpha _n, h(x_{x_{n+1}})\}$. Recently, Colao and Marino (Fixed Point Theory Appl 2015:39, 2015) have proved both weak and strong convergence theorems when *C* is a strictly convex set and *T* is an inward mapping. Meanwhile, they proposed a open question for a countable family of non-self nonexpansive mappings. In this article, authors will give an answer and will prove the further generalized results with the examples to support them.

## Background

Let *C* be a closed, convex and nonempty subset of a Hilbert space *H* and let $T : C \rightarrow H$ be a non-expansive mapping such that the fixed point set $F(T) := \{x \in C : Tx = x\}$ is nonempty. If *T* is a self-mapping, for $x_0\in C$ the Mann iterative scheme

1 $$x_{n+1}=\alpha _nx_n+(1-\alpha _n)Tx_n, \ \ \ n=0,1,2, \ldots$$

has been studied in an impressive amount of papers (see Chidume 2009 and the references therein) in the last decades and it is often called segmenting Mann (1953), Groetsch (1972), Hicks and Kubicek (1977) or Krasnoselskii–Mann (e.g., Edelstein and OBrien 1978; Hillam 1975) iteration. A general result on algorithm (1) is due to Reich (1979) and states that the sequence $\{x_n\}$ weakly converges to a fixed point of the operator *T* under the following assumptions:

$$(C_1)$$
$$T:C\rightarrow C$$;
$$(C_2)$$
$$\sum _{n=0}^{\infty }\alpha _n(1-\alpha _n)=\infty$$.

Many authors are interested in lowering condition $(C_1)$ by allowing *T* to be non-self at the price of strengthening the requirements on the sequence $\{\alpha _n\}$ and on the set *C*.

Historically, the inward condition and its generalizations were widely used to prove convergence results for both implicit (Xu and Yin 1995; Xu 1997; Marino and Trombetta 1992; Takahashi and Kim 1998) and explicit (see, e.g., Chidume 2009; Song and Chen 2006; Song and Cho 2009; Zhou and Wang 2014) algorithms. However, we point out that the explicit case was only studied in conjunction with processes involving the calculation of a projection or a retraction $P : H \rightarrow C$ at each step. As an example, in Song and Chen (2006), the following algorithm is studied:

$$x_{n+1}=P(\alpha _n f(x_n)+(1-\alpha _n)Tx_n)$$

where $T : C \rightarrow H$ satisfies the weakly inward condition, *f* is a contraction and $P : H \rightarrow C$ is a non-expansive retraction. However, in many real world applications, the process of calculating *P* can be a resource consumption task and it may require an approximating algorithm by itself, even in the case when *P* is the nearest point projection.

Recently, Colao and Marino (2015) introduced a new search strategy for the coefficients $\{\alpha _n\}$ and they have proved that the Krasnoselskii–Mann algorithm (1) is well defined for this particular choice of the sequence $\{\alpha _n\}$. Also they have proved both weak and strong convergence results for the above algorithm (1) when *C* is a strictly convex set and *T* is inward.

For a closed and convex set *C* and a map $T : C \rightarrow H$, we define a mapping $h : C \rightarrow R$ as

2 $$h(x)=\inf \{\lambda \ge 0 : \lambda x+(1-\lambda )Tx \in C\}$$

Note that the above quantity is a minimum since *C* is closed. The following lemma is useful which has been proved in Colao and Marino (2015).

### **Lemma VG**

(Colao and Marino 2015) *LetCbe a nonempty, closed and convex set, let*$T : C \rightarrow H$*be a mapping and define*$h : C \rightarrow R$*as in (2)*. *Then the following properties hold*:

$$(P_1)$$
*for any*$x \in C, h(x) \in [0, 1]$*and*$h(x) = 0$*if and only if*$Tx \in C$;
$$(P_2)$$
*for any*$x\in C$*and any*$\alpha \in [h(x), 1], \alpha x+(1-\alpha )Tx \in C;$
$$(P_3)$$
*ifTis an inward mapping, then*$h(x) < 1$*for any*$x \in C$;
$$(P_4)$$
*whenever*$$Tx \overline{\in } C, h(x)x+(1-h(x))Tx \in \partial C.$$

The following is a main result of Colao and Marino (2015).

### **Theorem VG**

(Colao and Marino 2015) *LetCbe a convex, closed and nonempty subset of a Hilbert spaceHand let*$T : C \rightarrow H$*be a mapping. Then the algorithm*

3 $${\left\{ \begin{array}{ll} x_0 \in C , \\ \alpha _0=\max \left\{ \frac{1}{2}, h(x_0),\right\} \\ x_{n+1}=\alpha _nx_n+(1-\alpha _n)Tx_n,\\ \alpha _{n+1}=\max \{\alpha _n, h(x_{n+1})\} \end{array}\right. }$$

*is well defined. If we further assume thatCis strictly convex andTis a non-expansive mapping, which satisfies the inward condition* (2) *and such that*$F(T)\ne \emptyset$. *Then*$\{x_n\}$*weakly converges to a point*$p \in F(T)$. *Moreover, if*$\sum _{n=0}^{\infty }(1-\alpha _n)<+\infty$, *then the convergence is strong*.

Meanwhile, Colao and Marino presented the following open question.

*Open question VG* (Colao and Marino 2015) Under which assumptions can algorithm (2) be adapted to produce a converging sequence to a common fixed point for a family of mappings ? In other words, does the algorithm

4 $${\left\{ \begin{array}{ll} x_0 \in C , \\ \alpha _0=\max \left\{ \frac{1}{2}, h_0(x_0),\right\} \\ x_{n+1}=\alpha _nx_n+(1-\alpha _n)T_nx_n,\\ \alpha _{n+1}=\max \{\alpha _n, h_{n+1}(x_{n+1})\} \end{array}\right.}$$

converge to a common fixed point of the family $\{T_n\}$, where

$$\begin{aligned} h_n(x)=\inf \{\lambda \ge 0 : \lambda x+(1-\lambda )T_nx \in C\} \end{aligned}$$

and under suitable hypotheses ?

In this paper, we will give a determinate answer for above open question *VG* and will give the further generalized results.

## The answer for the open question and main result

The following notions will be used in this paper. Of course, these notions have been also presented in the paper of Colao and Marino (2015).

### **Definition 1**

A map $T : C \rightarrow H$ is said to be inward (or to satisfy the inward condition) if, for any $x \in C$, it holds

$$Tx \in I_C(x)=\{x+c(u-x), c\ge 0, \ u \in C\}$$

We refer to Kirk and Sims (2001) for a comprehensive survey on the properties of the inward mappings.

### **Definition 2**

A set $C \subset H$ is said to be strictly convex if it is convex and with the property that $x, y \in \partial C$ and $t \in (0, 1)$ implies that $tx + (1 - t)y \in C^0$. In other words, if the boundary $\partial C$ does not contain any segment. Where $\partial C$ is the boundary of *C* and $C^0$ is the interior of *C*.

### **Definition 3**

A sequence $\{y_n\} \subset C$ is Fejér-monotone with respect to a set $D \subset C$ if, for any element $y \in D,$

$$\begin{aligned} \Vert y_{n+1}-p\Vert \le \Vert y_n-p\Vert, \quad \forall \ n\ge 1 \end{aligned}$$

In order to clearly answer the open question *VG*, we give the following notions.

### **Definition 4**

Let *D*, *C* be two closed and convex nonempty sets in a Hilbert space *H* and $D\subset C$. For any sequence $\{x_n\}\subset C$, if $\{x_n\}$ converges strongly to an element $x^* \in \partial C \setminus D, \ x_n\ne x^*$ implies that $\{x_n\}$ is not Fej$\acute{e}$r-monotone with respect to the set $D \subset C$, we called that, the pair (*D*, *C*) satisfies *S*-condition.

### **Definition 5**

Let $\{T_n\}$ be sequence of mappings from *H* into itself with nonempty common fixed point set $F=\cap _{n=1}^{\infty }F(T_n)$. The $\{T_n\}$ is said to be uniformly weakly closed if for any convergent sequence $\{z_n\} \subset C$ such that $\Vert T_nz_n-z_n\Vert \rightarrow 0$ as $n\rightarrow \infty$, the weak cluster points of $\{z_n\}$ belong to *F*.

### **Lemma 6**

(Reich 1979) *LetXbe a uniformly convex Banach space*, $\{x_n\}, \{y_n\}\subset X$*be two sequences, if there exists a constant*$d\ge 0$*such that*

$$\begin{aligned}&\limsup _{n\rightarrow \infty }\Vert x_n\Vert \le d, \quad \limsup _{n\rightarrow \infty }\Vert y_n\Vert \le d,\\&\lim _{n\rightarrow \infty }\Vert t_nx_n+(1-t_n)y_n\Vert =d, \end{aligned}$$

*the*$\lim _{n\rightarrow \infty }\Vert x_n-y_n\Vert =0$, *where*$t_n \in [a,b]\subset (0,1)$*anda*, *bare two constants*.

The following theorem is main result which is also a answer to the open question of Colao and Marino.

### **Theorem 7**

*LetCbe a convex, closed and nonempty subset of a Hilbert spaceHand let*$\{T_n\}_{n=0}^{\infty }: C \rightarrow H$*be a uniformly weakly closed countable family of non-self nonexpansive mappings. Then the algorithm (4) is well defined. Assume thatCis strictly convex and each*$T_n$*satisfies the inward condition and such that*$F=\cap _{n=0}^{\infty }F(T_n)\ne \emptyset$. *Then the following conclusions hold*:

1. *if there exist*$a,b \in (0,1)$*such that*$\alpha _n \in [a,b]$*for all*$n\ge 0$, *the*$\{x_n\}$*weakly converges to a common fixed point*$p \in F$.
2. *if*$\sum _{n=0}^{\infty }(1-\alpha _n)<+\infty$, *and* (*F*, *C*) *satisfiesS*-*condition, the*$\{x_n\}$*converges strongly to a common fixed point*$p \in F$.

### *Proof*

(1) for any $p\in F$ we have

$$\begin{aligned} \Vert x_{n+1}-p\Vert&= \Vert \alpha _nx_n+(1-\alpha _n)T_nx_n-p\Vert \\\le\;& \alpha _n\Vert x_n-p\Vert +(1-\alpha _n)\Vert T_nx_n-p\Vert \\\le\; & \alpha _n\Vert x_n-p\Vert +(1-\alpha _n)\Vert x_n-p\Vert \\=\; & \Vert x_n-p\Vert . \end{aligned}$$

Therefore there exists a constant *d* such that

$$\lim _{n\rightarrow \infty }\Vert x_n-p\Vert =d.$$

and

$$\begin{aligned} \limsup _{k\rightarrow \infty }\Vert T_{n}x_{n}-p\Vert \le d. \end{aligned}$$

Moreover

$$\begin{aligned}&\Vert \alpha _{n}(x_{n}-p)+(1-\alpha _{n})(T_{n}x_{n}-p)\Vert \\&\quad =\Vert \alpha _{n}x_{n}+(1-\alpha _{n})T_{n}x_{n}-p\Vert =\Vert x_{n+1}-p\Vert \rightarrow d \end{aligned}$$

as $k\rightarrow \infty$. By using Lemma 6, we have

$$\begin{aligned} \lim _{k\rightarrow \infty }\Vert T_{n}x_{n}-x_{n}\Vert = 0. \end{aligned}$$

Since $\{x_{n}\}$ is bounded, there exists a subsequence $\{x_{n_k}\}$ such that it converges weakly to a element $x^* \in C.$ Since $\{T_n\}$ is uniformly weakly closed, it follows $x^* \in F$. Next we claim that $\{x_n\}$ converges weakly to this element $x^*$. If not, there exists a subsequence $\{x_{m_k}\}$ does not converges weakly to $x^*$, then there must exist a subsequence $\{x_{m_{k_i}}\}$ such that it converges weakly to another element $y^*\ne x^*$ and $y^* \in F.$ Hilbert space *H* satisfies Opial’s condition, we have

$$\begin{aligned} \lim _{n\rightarrow \infty }\Vert x_n-x^*\Vert&= \lim _{k\rightarrow \infty }\Vert x_{n_k}-x^*\Vert \\< \lim _{k\rightarrow \infty }\Vert x_{n_k}-y^*\Vert&= \lim _{k\rightarrow \infty }\Vert x_{m_k}-y^*\Vert \\ < \lim _{n\rightarrow \infty }\Vert x_{m_k}-x^*\Vert&= \lim _{n\rightarrow \infty }\Vert x_n-x^*\Vert . \end{aligned}$$

This is a contradiction. This show that $\{x_n\}$ converges weakly to this element $x^*\in F$.

(2) Since

$$\begin{aligned} \sum _{n=0}^{\infty }(1-\alpha _n)<+\infty \end{aligned}$$

and by the boundedness of $\{x_n\}$ and $\{Tx_n\}$, it is promptly obtained that

$$\begin{aligned} \sum _{n=0}^{\infty }\Vert x_{n+1}-x_n\Vert <+\infty \end{aligned}$$

i.e., $\{x_n\}$ is a strongly Cauchy sequence and hence $x_n\rightarrow x^* \in C$. If there exists a natural number *N* such that $n>N$ implies $x_n=x^*$, the conclusion is right. In the other case, note that each $T_n$ satisfies the inward condition. Then, by applying properties $(P_2)$ and $(P_3)$ from Lemma *VG*, we obtain that $h_n(x^*)<1$ for all $n\ge 0$ and that for any $\mu _n \in (h_n(x^*),1)$ it holds

$$\mu _nx^*+(1-\mu _n)T_nx^* \in C.$$

On the other hand, we observe that since $\lim _{n\rightarrow \infty }\alpha _n=1$ and since $\alpha _{n+1}=\max \{\alpha _n, h_{n+1}(x_{n+1})\}$ holds, it follows that we can choose a sub-sequence $\{x_{n_k}\}$ with the property that $h_{n_k}(x_{n_k})$ is non-decreasing and $\lim _{k\rightarrow \infty }h_{n_k}(x_{n_k})=1$. Hence

5 $$\begin{aligned} \lim _{k\rightarrow \infty } \left( \frac{k}{k+1}h_{n_k}(x_{n_k})x_{n_k} +\left( 1-\frac{k}{k+1}h_{n_k}(x_{n_k})\right) T_{n_k}x_{n_k}\right) =x^*. \end{aligned}$$

On the other hand

$$\begin{aligned} \frac{k}{k+1}h_{n_k}(x_{n_k})x_{n_k}+ \left( 1-\frac{k}{k+1}h_{n_k}(x_{n_k})\right) T_{n_k}x_{n_k} \overline{\in } C, \end{aligned}$$

this together with (5) implies $x^* \in\, \partial \, C$. Since $\{x_n\}$ is Fej$\acute{e}$r-monotone with respect to a set $F \subset C$, the *S*-condition implies $x^* \in F$. This completes the proof. $\square$

### *Remark*

The proved theorem is a partial answer to the open question that it is not completely satisfactory. In fact the assumption that can not approach to 1, imposes a restriction a priori on $\alpha _n$. It remains an open question whether the thesis holds without assumptions a priori on.

### **Definition 8**

A mapping $T: C \rightarrow H$ is said to be quasi-nonexpansive, if the fixed point set *F*(*T*) is nonempty and

$$\Vert Tx-p\Vert \le \Vert x-p\Vert , \ \forall \ x \in C, \ p\in F(T).$$

By using the same method of proof as in Theorem 7, we can prove Theorem 7 is still right for quasi-nonexpansive mappings. Therefore, we can get the further generalized result as follows.

### **Theorem 9**

*LetCbe a convex, closed and nonempty subset of a Hilbert spaceHand let*$\{T_n\}_{n=0}^{\infty }: C \rightarrow H$*be a uniformly weakly closed countable family of non-self quasi-nonexpansive mappings. Then the algorithm (4) is well defined. Assume thatCis strictly convex and each*$T_n$*satisfies the inward condition and such that*$F=\cap _{n=0}^{\infty }F(T_n)\ne \emptyset$. *Then the following conclusions hold*:

1. *if there exist*$a,b \in (0,1)$*such that*$\alpha _n \in [a,b]$*for all*$n\ge 0$, *the*$\{x_n\}$*weakly converges to a common fixed point*$p \in F$.
2. *if*$\sum _{n=0}^{\infty }(1-\alpha _n)<+\infty$, *and* (*F*, *C*) *satisfiesS*-*condition, the*$\{x_n\}$*converges strongly to a common fixed point*$p \in F$.

## Examples

Let *A* be a multi-valued operator from *H* into it-self with domain $D(A)=\{z\in E: Az\ne \emptyset \}$ and range $R(A)=\{z\in E: z\in D(A)\}$. An operator *A* is said to be monotone if

$$\langle x_1-x_2, y_1-y_2 \rangle \ge 0$$

for each $x_1, x_2 \in D(A)$ and $y_1\in Ax_1, y_2\in Ax_2$. A monotone operator *A* is said to be maximal if it’s graph $G(A)=\{(x, y) : y \in Ax\}$ is not properly contained in the graph of any other monotone operator. We know that if *A* is a maximal monotone operator, then $A^{-1}0$ is closed and convex. The following result is also well-known.

### **Theorem 10**

(Rockafellar Rockafellar 1970). *LetHbe a Hilbert space and letAbe a monotone operator fromHinto it-self. ThenAis maximal if and only if*$R(I + rA)=H$. *for all*$r>0$, *whereIis the identity operator*.

Let *H* be a Hilbert space, and let *A* be a maximal monotone operator from *H* into it-self. Using Theorem 10, we obtain that for every $r>0$ and $x\in H$, there exists a unique $x_r$ such that

$$x\in x_r+rAx_r.$$

Then we can define a single valued mapping $J_r :H \rightarrow D(A)$ by $J_r = (I + rA)^{-1}$ and such a $J_r$ is called the resolvent of *A*. We know that $A^{-1}0=F(J_r)$ for all $r > 0$, see Takahashi (2000a, b) for more details.

### *Example 11*

Let *H* be a Hilbert space, let *A* be a maximal monotone operator from *H* into it-self such that $A^{-1}0 \ne \emptyset$, let $J_r$ be the resolvent of *A*, where $r>0$. For $r_n>0, \ \limsup _{n\rightarrow \infty } r_n>0$, the $\{J_{r_n}\}_{n=0}^{\infty }$ is an uniformly weakly closed countable family of quasi-nonexpansive mappings.

### *Proof*

For any $p\in \bigcap _{n=0}^{\infty }F(J_{r_n})=A^{-1}0 \ne \emptyset ,\ w \in H$, from the monotonicity of *A*, we have

$$\begin{aligned} \Vert p- J_{r_n}w\Vert ^2&=\Vert p\Vert ^2-2\langle p, J_{r_n}w\rangle +\Vert J_{r_n}w\Vert ^2\\&=\Vert p\Vert ^2+2\langle p, w-J_{r_n}w-w\rangle +\Vert J_{r_n}w\Vert ^2\\&=\Vert p\Vert ^2+2\langle p, w-J_{r_n}w\rangle -2\langle p, w\rangle +\Vert J_{r_n}w\Vert ^2\\&=\Vert p\Vert ^2-2\langle J_{r_n}w-p-J_{r_n}w, w-J_{r_n}w-w\rangle -2\langle p, w\rangle +\Vert J_{r_n}w\Vert ^2\\&=\Vert p\Vert ^2-2\langle J_{r_n}w-p, w-J_{r_n}w-w\rangle \\ {}&\ \ \ \ +2\langle J_{r_n}w, w-J_{r_n}w\rangle -2\langle p, w\rangle +\Vert J_{r_n}w\Vert ^2\\&\le \Vert p\Vert ^2+2\langle J_{r_n}w, w-J_{r_n}w\rangle -2\langle p, w\rangle +\Vert J_{r_n}w\Vert ^2\\&=\Vert p\Vert ^2-2\langle p, w\rangle +\Vert w\Vert ^2-\Vert J_{r_n}w\Vert ^2+2\langle J_{r_n}w, w\rangle -\Vert w\Vert ^2\\&=\Vert p-w\Vert ^2-\Vert J_{r_n}w-w\Vert ^2\\&\le \Vert p-w\Vert ^2 \end{aligned}$$

for all $n\ge 0$. Let $\{z_n\}$ be a sequence in *H* such that $\lim _{n\rightarrow \infty }\Vert z_n-J_{r_n}z_n\Vert =0$. Let *q* be a weak cluster point of $\{z_n\}$, then there exists a subsequence $\{z_{n_k}\}\subset \{z_n\}$ such that $\{z_{n_k}\}$ converges weakly to *q*. In this case, we have

$$\begin{aligned} \frac{1}{r_{n_k}}(z_{n_k}-J_{r_{n_k}}z_{n_k})\rightarrow 0. \end{aligned}$$

It follows from

$$\begin{aligned} \frac{1}{r_{n_k}}(z_{n_k}-J_{r_{n_k}}z_{n_k})\in AJ_{r_{n_k}}z_{n_k} \end{aligned}$$

and the monotonicity of *A* that

$$\begin{aligned} \left\langle w-J_{r_{n_k}}z_{n_k}, w^*-\frac{1}{r_{n_k}}(z_{n_k}-J_{r_{n_k}}z_{n_k})\right\rangle \ge 0 \end{aligned}$$

for all $w\in D(A)$ and $w^*\in Aw$. Letting $k\rightarrow \infty$, we have $\langle w-q, w^*\rangle \ge 0$ for all $w\in D(A)$ and $w^*\in Aw$. Therefore from the maximality of *A*, we obtain $q\in A^{-1}0$ and hence $q \in \cap _{n=1}^{\infty }F(J_{r_n})$. Therefore, $\{J_{r_n}\}_{n=1}^{\infty }$ is an uniformly weakly closed countable family of quasi-nonexpansive mappings. This completes the proof. $\square$

### *Example 12*

Let $$H=R^2$$,

$$\begin{aligned} D= & {} \{(x,y) \in H: x^2+y^2\le 1\}\\ C= & {} \{(x,y) \in H: x^2+y^2\le 2\}. \end{aligned}$$

It is obvious that, (*D*, *C*) satisfies *S*-condition.

### *Example 13*

Let $$H=R^2$$,

$$\begin{aligned} D= & {} \left\{ (x,y) \in H: \frac{x^2}{2}+\frac{y^2}{3}\le 1\right\} \\ C= & {} \left\{ (x,y) \in H:\frac{x^2}{2}+\frac{y^2}{3}\le 2\right\} . \end{aligned}$$

It is obvious that, (*D*, *C*) satisfies *S*-condition.

## Conclusions

Let *C* be a convex, closed and nonempty subset of a Hilbert space *H* and let $\{T_n\}_{n=0}^{\infty }: C \rightarrow H$ be a uniformly weakly closed countable family of non-self nonexpansive mappings. Then the algorithm (4) is well defined. Assume that *C* is strictly convex and each $T_n$ satisfies the inward condition and such that $F=\cap _{n=0}^{\infty }F(T_n)\ne \emptyset$. Then the following conclusions hold:

1. if there exist $a,b \in (0,1)$ such that $\alpha _n \in [a,b]$ for all $n\ge 0$, the $\{x_n\}$ weakly converges to a common fixed point $p \in F$.
2. if $\sum _{n=0}^{\infty }(1-\alpha _n)<+\infty$, and (*F*, *C*) satisfies *S*-condition, the $\{x_n\}$ converges strongly to a common fixed point $p \in F$.
